# Robotic versus laparoscopic surgery in hepatobiliary procedures: a scoping review

**DOI:** 10.1186/s12893-026-03907-y

**Published:** 2026-07-04

**Authors:** Jerónimo Cárdenas Montoya, Mariana González Garcés, Mario Andrés Torres Torres, Erwin Hernando Hernández Rincón, Diego Orlando Sierra Barbosa

**Affiliations:** 1 Primary Care Physician, School of Medicine, Universidad de La Sabana, Chía, Colombia; 2 Department of Family Medicine and Public Health, Universidad de La Sabana, University Campus Puente del Común, Km 7, Autopista Norte, Chía, Colombia; 3 Department of General Surgery, Universidad de La Saban, Chía, Colombia

**Keywords:** Hepatobiliary surgery, Robotic surgery, Laparoscopic surgery, Minimally invasive surgery, Procedure-stratified analysis, Bile duct injury

## Abstract

**Background:**

Robotic platforms have expanded the technical capabilities of minimally invasive hepatobiliary surgery. However, their clinical value compared with laparoscopy remains heterogeneous and appears to vary according to procedural complexity, anatomical constraints, and institutional experience.

**Methods:**

A procedure-stratified scoping review was conducted in accordance with the Preferred Reporting Items for Systematic Reviews and Meta-Analyses extension for Scoping Reviews and the Joanna Briggs Institute Manual for Evidence Synthesis. PubMed, Scopus, and Web of Science were searched for comparative studies published between 2019 and 2025 evaluating robotic versus laparoscopic hepatobiliary surgery. Evidence was synthesized using a procedure-specific analytical framework to account for heterogeneity in technical complexity and outcome reporting.

**Results:**

Seventy studies met inclusion criteria, the majority of which were retrospective. In technically demanding procedures, particularly major hepatectomy and resections of posterosuperior liver segments, robotic surgery was more frequently associated with lower intraoperative blood loss and reduced conversion to open surgery. Operative time was generally longer for robotic procedures across most indications. Overall perioperative morbidity, mortality, and early oncological outcomes were comparable between robotic and laparoscopic approaches. Postoperative recovery outcomes were inconsistently reported and heterogeneous, while procedural costs were consistently higher for robotic surgery.

**Conclusions:**

Robotic hepatobiliary surgery may offer context-dependent advantages in anatomically complex procedures, particularly in settings with appropriate surgical expertise. However, given the predominance of retrospective and heterogeneous evidence, these findings should be interpreted cautiously. Overall, robotic and laparoscopic approaches demonstrate comparable safety and effectiveness across most indications. These findings support a procedure-based and context-sensitive integration of robotic technology rather than universal adoption.

**Supplementary Information:**

The online version contains supplementary material available at https://doi.org/10.1186/s12893-026-03907-y.

## Introduction

Minimally invasive surgery has become widely adopted in hepatobiliary surgery and contributes substantially to contemporary surgical practice, demonstrating improved perioperative outcomes such as reduced postoperative pain, shorter length of hospital stay, and faster functional recovery compared with open approaches [1–5]. However, comparative evaluation of robotic and laparoscopic approaches remains challenging because of substantial heterogeneity across procedures, anatomical complexity, and outcome reporting. Most studies continue to evaluate hepatobiliary surgery as a single entity, potentially obscuring clinically relevant differences between distinct procedures. In this context, robotic-assisted surgery has emerged as an extension of minimally invasive techniques, particularly in anatomically complex operative settings [6–10].

In hepatobiliary surgery, robotic platforms have been described in relation to three-dimensional visualization, articulated instrumentation, tremor filtration, and ergonomic configuration. These characteristics have been most frequently discussed in procedures requiring vascular control, complex parenchymal transection, or intracorporeal suturing, such as major hepatectomy, posterosuperior segment resections, and biliary reconstruction [5, 6, 10]. However, the clinical relevance of these reported differences remains debated because of heterogeneity in study design, procedural selection, and outcome reporting [11–13].

The available literature comparing robotic and laparoscopic hepatobiliary surgery remains heterogeneous. Several systematic reviews and meta analyses have reported variable findings regarding operative time, intraoperative blood loss, conversion to open surgery, and perioperative morbidity [3, 5, 6, 11, 13]. Importantly, many analyses have pooled diverse hepatobiliary procedures into broad categories, potentially limiting interpretation of procedure specific findings relevant to operative planning and clinical decision making [14].

Procedure complexity represents an important determinant of outcomes in hepatobiliary surgery, although definitions of complexity remain inconsistent across studies. Difficulty scoring systems such as the Iwate criteria have been proposed to stratify minimally invasive liver resections according to anatomical location, extent of resection, proximity to major vessels, and liver function [15]. Additional robotic-specific complexity frameworks have also been described in high volume hepatobiliary centres [16]. However, standardized complexity scoring systems remain inconsistently applied across comparative studies.

Economic considerations further complicate interpretation of comparative findings. Most available studies focus on procedural costs without accounting for broader institutional or healthcare system factors, and the majority of published evidence originates from high resource settings [17–19].

Given these limitations, a procedure stratified synthesis of the available literature is needed. Therefore, the objective of this scoping review was to systematically map and synthesize studies comparing robotic and laparoscopic approaches in hepatobiliary surgery, with stratification according to procedure type, technical complexity, and outcome domain.

## Methodology

### Study design

A scoping review was conducted in accordance with the Preferred Reporting Items for Systematic Reviews and Meta Analyses extension for Scoping Reviews [20] and the Joanna Briggs Institute Manual for Evidence Synthesis [21]. This methodological approach was selected to systematically map the breadth, heterogeneity, and limitations of the available evidence comparing laparoscopic and robotic approaches in hepatobiliary surgery, rather than to generate pooled effect estimates. Scoping review methodology was considered appropriate given the diversity of study designs, procedures, outcome definitions, and analytical approaches across the existing literature.

The study protocol was prospectively registered on the Open Science Framework to ensure methodological transparency and reproducibility and is publicly available at https://osf.io/e3c4y/.

The review was guided by the Population-Concept-Context framework [22]. The primary research question was: what evidence is currently available regarding differences in perioperative, postoperative recovery, oncological, and cost related outcomes between laparoscopic and robotic surgery in adult patients undergoing hepatobiliary procedures? Given the substantial variability in anatomical complexity and technical demands across hepatobiliary interventions, a predefined analytical objective was to synthesize findings in a disaggregated and procedure specific manner.

### Information sources and search strategy

A comprehensive and systematic literature search was performed in PubMed, Scopus, and Web of Science. Eligible studies published between January 2019 and September 2025 were considered. The search strategy combined controlled vocabulary terms and free text keywords related to laparoscopic surgery, robotic assisted surgery, and hepatobiliary procedures, including hepatectomy, cholecystectomy, and biliary reconstruction, together with outcome related terms such as operative time, blood loss, conversion to open surgery, postoperative complications, length of hospital stay, readmission, and quality of life.

Search strategies were adapted to the syntax and indexing of each database to maximize sensitivity and minimize retrieval bias. All retrieved records were imported into Rayyan for duplicate removal and initial screening. Full search strategies for each database are provided in the Supplementary Material to ensure reproducibility and transparency.

### Eligibility criteria

Eligible studies included original clinical research with prospective, retrospective, or multicentre designs, as well as systematic reviews and meta analyses, that directly compared laparoscopic and robotic approaches in adult patients aged eighteen years or older undergoing hepatobiliary surgery. Procedures of interest included anatomical and non-anatomical hepatectomy, complex cholecystectomy, and biliary tract surgery.

To be included, studies were required to report at least one perioperative outcome, such as operative time, blood loss, transfusion requirements, conversion to open surgery, postoperative complications, or mortality, or at least one postoperative recovery outcome, including length of hospital stay, postoperative pain, analgesic consumption, readmission, or quality of life measures.

Studies involving pediatric populations, animal models, single case reports, editorials, commentaries, or letters were excluded. Studies lacking a direct comparative design between laparoscopic and robotic approaches were also excluded. Publications not written in English or Spanish were excluded.

### Study selection

**Fig. 1:**
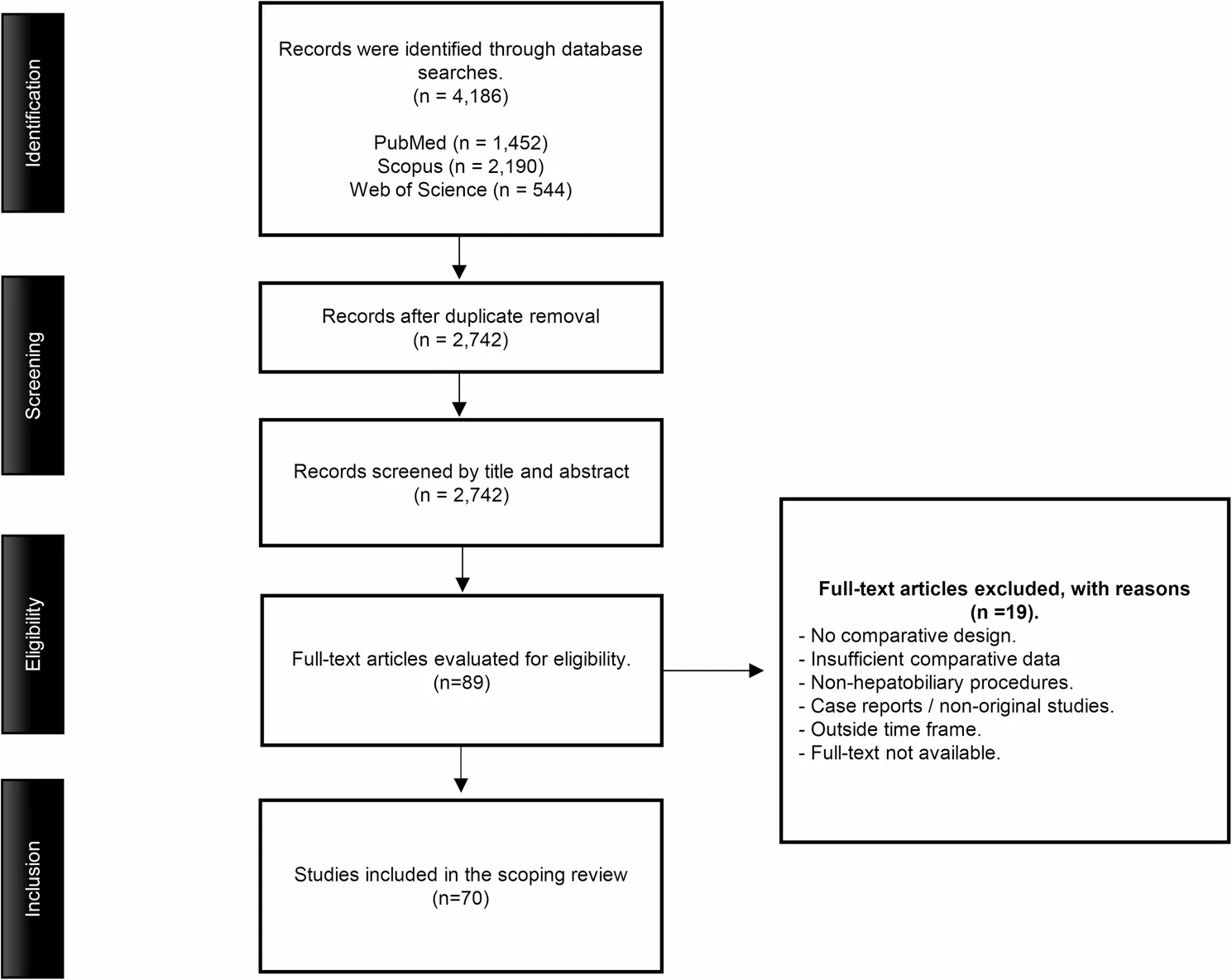
Prisma-ScR

Two reviewers independently screened titles, abstracts, and full text articles according to predefined eligibility criteria. Disagreements were resolved through discussion and consensus. The study selection process is summarized in Fig. 1.

### Data extraction

Data extraction was performed using a standardized electronic data collection form developed in Microsoft Excel. Extracted variables included author, year of publication, country, study design, sample size, type of hepatobiliary procedure, definitions of procedural complexity when reported, perioperative outcomes, postoperative recovery outcomes, oncological variables, cost related data, learning curve indicators, and reported study limitations.

Two reviewers independently performed data extraction to enhance data accuracy and reliability. Disagreements were resolved through discussion and consensus.

### Data synthesis and analytical framework

Rather than treating hepatobiliary surgery as a single homogeneous entity, findings were synthesized narratively using a procedure stratified analytical framework. Interpretation focused on predefined clinical scenarios, including minor hepatectomy, major hepatectomy, posterosuperior segment resections, complex cholecystectomy, and biliary reconstruction. This approach was chosen to account for heterogeneity across procedures and facilitate procedure specific interpretation of findings.

Although validated surgical difficulty scoring systems such as the Iwate criteria and more recent complexity frameworks have been proposed, their application was inconsistent across the included studies. Consequently, procedural stratification was employed as a descriptive approach to procedural complexity, while explicitly acknowledging that this approach does not substitute for standardized complexity scoring systems.

Given the inclusion of both primary comparative studies and secondary evidence such as systematic reviews and meta analyses, overlap between evidence sources was minimized by using secondary studies primarily for contextual interpretation rather than as independent data points. In addition, no attempt was made to derive pooled estimates or infer comparative effectiveness, in line with the exploratory nature of scoping review methodology.

### Methodological and data source considerations

Given the predominance of retrospective study designs, methodological limitations frequently described in the included literature were considered during interpretation, including selection bias, confounding by surgeon experience, institutional case volume, and variability in the use of statistical adjustment methods such as propensity score matching. In addition, limitations related to the use of administrative databases and clinical registries were acknowledged, including potential misclassification of surgical approach and incomplete capture of intraoperative variables such as conversion to open surgery.

In accordance with scoping review methodology, a formal risk of bias assessment was not performed. Instead, emphasis was placed on mapping variability and reporting patterns across the existing literature.

### Ethical considerations

This study analyzed previously published data and did not involve human participants or identifiable personal information. Therefore, approval from an ethics committee was not required.

## Results

### Study characteristics and geographical distribution

Seventy studies fulfilled the predefined eligibility criteria and were included in the scoping review. Most included publications were published after 2021, reflecting the increasing volume of literature evaluating minimally invasive approaches in hepatobiliary surgery.

**Fig. 2:**
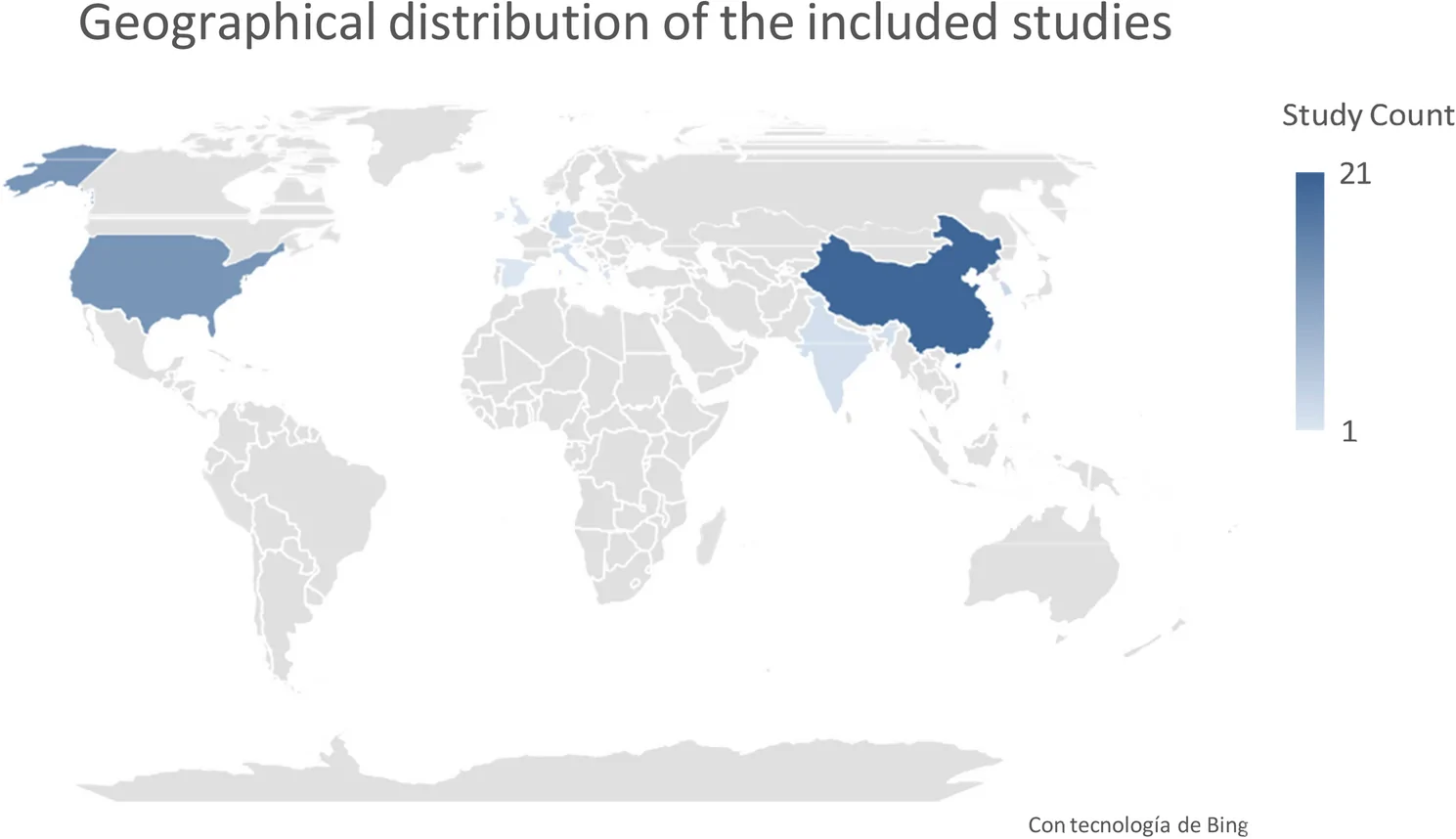
Geographical distribution of the studies included. The geographical distribution of the included studies revealed a clear predominance in China (*n* = 20) and the United States (*n* = 13), which together contributed a significant proportion of the available evidence. Additional contributions came from Germany (*n* = 3), South Korea (*n* = 3), and Italy (*n* = 2). Other countries with less representation included India (*n* = 2), the United Kingdom (*n* = 1), Spain (*n* = 1), Austria (*n* = 1), Greece (*n* = 1), Ireland (*n* = 1), and Taiwan (*n* = 1). In addition, 21 international or multicentre studies were identified, demonstrating a growing collaborative effort to evaluate the outcomes of laparoscopic and robotic surgery in hepatobiliary pathology

The included studies originated predominantly from Asia, Europe, and North America, with China and the United States contributing the largest proportion of publications (Fig. 2). Several investigations were conducted as multicentre or international collaborations, including propensity score matched cohorts and registry-based analyses, representing a broad range of contemporary surgical settings [23–29]. However, robotic experience was most frequently reported in high volume centres, whereas studies from low volume or early adoption settings remained limited.

Regarding study design, most included studies were retrospective comparative analyses. Systematic reviews and meta analyses were also frequently identified, particularly in studies evaluating hepatectomy and cholecystectomy outcomes, whereas prospective and randomized evidence remained limited [30–34]. Among retrospective studies, the use of statistical adjustment methods such as propensity score matching varied substantially, and several studies relied on unadjusted comparisons.

### Perioperative outcomes

Across studies evaluating hepatectomy, several reports described lower intraoperative blood loss and fewer conversions to open surgery in robotic cohorts, particularly in major hepatectomy and posterosuperior segment resections [23, 24, 27, 35–39]. These findings were described in both multicentre cohorts and procedure specific evidence syntheses [31, 32]. However, the magnitude and consistency of reported findings varied across studies and institutional settings.

Longer operative times in robotic procedures were frequently reported across multiple comparative series, including studies involving major hepatectomy and broader minimally invasive liver surgery series [28, 40–44]. Perioperative morbidity and mortality outcomes were variably reported, with most studies describing similar perioperative profiles across approaches.

A summary of reported perioperative findings, including operative time, estimated blood loss, transfusion requirements, conversion rates, and perioperative morbidity, is presented in Table 1.

**Table 1:** Procedure-stratified summary of reported perioperative findings in robotic and laparoscopic hepatobiliary surgery

Procedure category	Outcome domain	Reported findings across studies	Contextual considerations	Key references*
Minor hepatectomy	Operative time	Similar or slightly longer operative times reported in robotic procedures	Differences were less consistent in lower-complexity resections	[11, 45–47]
	Blood loss	Comparable blood loss reported across approaches	Similar vascular control reported in anterolateral resections	[45, 46, 48]
	Conversion to open surgery	Low conversion rates reported in both approaches	Low baseline conversion rates across studies	[11, 47, 49]
	Overall morbidity	Similar perioperative morbidity reported	No consistent differences identified	[46, 47, 50]
Major hepatectomy	Operative time	Longer operative times frequently reported in robotic procedures	Case complexity and setup requirements were frequently discussed in included studies	[23, 51, 52]
	Blood loss	Several studies described lower blood loss in robotic cohorts	Findings more commonly described in complex resections	[23, 24, 27, 35]
	Conversion to open surgery	Lower conversion rates were reported in some robotic cohorts	Findings mainly reported in experienced centres	[23, 27, 35, 53]
	Major complications	Variable complication rates reported across studies	Surgeon and institutional experience were frequently discussed across studies	[23, 28, 51]
Posterosuperior segment resections	Technical exposure	Visualization and articulation features were frequently described in robotic procedures	Frequently reported in anatomically constrained fields	[27, 35, 54]
	Blood loss	Lower blood loss reported in several robotic series	Mainly described in technically demanding resections	[27, 54, 55]
	Conversion to open surgery	Reduced conversion rates described in selected robotic cohorts	Findings varied across institutional experiences	[35, 53, 54]
Complex cholecystectomy	Operative time	Longer operative times commonly reported in robotic procedures	Often associated with docking and setup requirements	[56–58]
	Conversion to open surgery	Variable conversion rates reported across studies	Frequently discussed in relation to surgeon experience	[56, 58, 59]
	Bile duct injury (BDI)	Inconsistent reporting of BDI outcomes across approaches	Frequently discussed in relation to learning curve	[56, 60, 61]
Biliary reconstruction	Technical feasibility	Robotic reconstruction was frequently described in studies involving intracorporeal suturing	Articulation and ergonomics commonly highlighted	[40, 62, 63]
	Anastomotic outcomes	Anastomotic outcomes inconsistently reported	Limited reporting of leaks and strictures	[62, 63]
Oncologic resections	R0 margins	Similar R0 resection rates reported across approaches	Oncological outcomes heterogeneously reported	[28, 64, 65]
	Lymphadenectomy	Similar lymphadenectomy findings described across approaches	Reporting varied substantially between studies	[28, 65, 66]

*References correspond to studies included in the scoping review

### Postoperative recovery outcomes

Postoperative recovery outcomes demonstrated substantial heterogeneity across included studies. Several reports described shorter hospital stay, earlier functional recovery, or lower postoperative pain scores in robotic cohorts, whereas other studies reported no consistent differences between robotic and laparoscopic approaches [18, 23, 67–74]. Similar variability was observed in systematic reviews and meta analyses evaluating postoperative recovery outcomes [34, 75, 76].

Differences in outcome definitions, perioperative care pathways, and measurement instruments were frequently described across studies. Few investigations employed standardized patient reported outcome measures, and reporting of analgesic use, pain scales, and recovery milestones remained inconsistent.

Postoperative recovery findings, including length of hospital stay, postoperative pain, analgesic use, and readmission outcomes, are summarized in Table 2.

**Table 2:** Procedural complexity and heterogeneity of complexity definitions across included studies

Procedure category	Reported sources of technical complexity	How complexity was defined in included studies	Use of validated complexity scores	Key references*
Minor hepatectomy	Limited parenchymal transection; anterolateral segments	Often defined according to extent of resection	Infrequently reported across included studies	[11, 45–47]
Major hepatectomy	Vascular inflow control; large transection surface	Definitions based on extent of resection	Iwate score reported in selected studies	[23, 24, 35, 51, 52]
Posterosuperior segment resections	Restricted exposure; proximity to diaphragm and major vessels	Definitions based on anatomical segment location	Iwate score frequently reported	[27, 35, 54, 55]
Complex cholecystectomy	Inflammation; adhesions; cirrhosis; prior surgery	Variable and frequently author-defined	No consistently applied validated complexity score identified	[56–59]
Biliary reconstruction	Intracorporeal suturing in deep operative fields	Procedure-based definitions	No validated complexity tool consistently reported	[40, 62, 63]
Oncologic resections	Tumor location; margin proximity; lymphadenectomy	Variable oncologic definitions across studies	Inconsistent use of staging systems	[28, 64, 65]

*References correspond to studies included in the scoping review

### Oncological outcomes, learning curve, and costs

Among studies evaluating malignant hepatobiliary disease, R0 resection rates and lymphadenectomy outcomes were reported as similar across approaches across robotic and laparoscopic approaches [28, 65, 66]. However, long term oncological outcomes, including recurrence and survival, were inconsistently reported across studies.

Several studies discussed learning curve patterns during robotic surgery adoption, most commonly describing decreases in operative time, blood loss, and conversion rates over time during later phases of institutional and surgeon experience [40, 64]. Learning curve reporting was most frequent in hepatectomy and cholecystectomy cohorts, although definitions of proficiency thresholds varied substantially across studies.

Procedure specific cost analyses frequently described higher procedural costs in robotic surgery compared with laparoscopy, primarily related to platform acquisition, maintenance, and disposable instrumentation [18, 19]. Formal cost effectiveness analyses and incremental cost effectiveness ratios were not identified among included studies.

Reported oncological findings, learning curve observations, cost related outcomes, and evidence limitations are summarized in Table 3.

**Table 3:** Reported themes related to oncological outcomes, learning curve, costs, postoperative recovery, and evidence limitations

Domain	Reported findings across included studies	Contextual observations	Key references*
Oncological outcomes	R0 margins and lymphadenectomy outcomes were generally reported as comparable across approaches; long-term oncological outcomes were inconsistently reported	Oncological reporting varied substantially across studies	[28, 64, 65, 77]
Learning curve and surgeon experience	Progressive reductions in operative time and conversion rates were reported during later adoption phases	Findings were frequently reported in high-volume centres and established robotic programs	[18, 56, 60, 73]
Cost-related outcomes	Higher procedural costs were frequently reported in robotic procedures	Economic analyses varied substantially across institutional settings	[17, 31, 78, 79]
Postoperative recovery (LOS, readmission)	Length of stay and readmission outcomes were variably reported across studies	Perioperative pathways differed substantially between institutions	[23, 28, 45, 46]
Postoperative pain and PROs	Pain and quality-of-life outcomes were heterogeneously defined	Reporting of validated PRO instruments remained limited	[16, 80, 81]
Bile duct injury (BDI)	BDI outcomes were infrequently reported across studies	Findings were frequently discussed in relation to learning curve and procedural complexity	[28, 64, 82]
Evidence base limitations	Included studies were predominantly retrospective and heterogeneous in methodology and reporting	Variability in adjustment methods and database granularity was frequently described	[23, 24, 83, 84]

*References correspond to studies included in the scoping review

### Data source and reporting limitations identified in included studies

Several studies based on administrative claims databases or large registries described limitations related to incomplete capture of intraoperative variables, including conversion to open surgery. Additionally, recent studies reported potential misclassification of surgical approach in insurance claims data, limiting interpretation of comparisons between robotic and laparoscopic procedures in some datasets [84].

These reporting limitations were frequently acknowledged across observational studies, particularly in relation to conversion rates, perioperative complications, and procedure classification.

## Discussion

This scoping review highlights the substantial heterogeneity within minimally invasive hepatobiliary surgery. By adopting a procedure stratified analytical framework, this review identified variability in reported perioperative and postoperative findings according to procedural complexity, anatomical constraints, surgeon experience, and institutional context [23, 24, 31]. Across the included literature, robotic and laparoscopic approaches were evaluated in a wide range of procedural settings, with differences in outcome reporting frequently associated with variation in case selection, centre volume, and methodological design.

### Perioperative outcomes and procedural complexity

Several included studies reported lower intraoperative blood loss and fewer conversions to open surgery in robotic cohorts undergoing technically demanding procedures, particularly major hepatectomy and posterosuperior segment resections [23, 24, 27, 32, 35, 85]. These findings were most frequently reported in studies conducted at high volume centres and in cohorts involving experienced robotic programs. Reported perioperative findings varied substantially across institutional settings and procedure types, emphasizing the importance of interpreting outcomes within the context of procedural complexity and study design.

From a technical perspective, several studies discussed platform characteristics such as three-dimensional visualization, articulated instrumentation, and ergonomic configuration in relation to complex hepatobiliary procedures. However, these interpretations remain descriptive and hypothesis-generating, as the available evidence is predominantly observational and subject to confounding by surgeon expertise, institutional experience, and case selection [23]. Emerging technologies such as intraoperative navigation and image-guided systems have also been described as potential adjuncts in technically demanding hepatobiliary procedures, particularly in anatomically constrained operative fields [78, 86].

Longer operative times in robotic procedures were frequently reported across multiple comparative series, including studies from experienced centres [28, 40]. Operative time findings varied according to procedure type, institutional workflow, and surgeon experience.

Perioperative morbidity and mortality outcomes were variably reported across studies, with several reports describing similar perioperative profiles between robotic and laparoscopic approaches [31, 67, 87]. Several studies also discussed the influence of surgeon experience, patient selection, and institutional factors on perioperative outcomes. Within this context, bile duct injury remains an important outcome measure in hepatobiliary surgery because of its association with postoperative morbidity, reintervention, and prolonged recovery [60].

Although this review used procedure stratification as a pragmatic proxy for surgical complexity, validated difficulty scoring systems such as the Iwate criteria and other complexity frameworks developed in robotic hepatobiliary surgery were inconsistently applied across included studies [15]. The absence of standardized complexity scoring limited cross study comparability and limited more granular interpretation of reported differences between approaches.

### Recovery and patient centred outcomes

Postoperative recovery outcomes demonstrated substantial heterogeneity across the included literature. Several studies described shorter hospital stay, earlier functional recovery, or lower postoperative pain scores in robotic cohorts, whereas others reported no consistent differences between robotic and laparoscopic approaches [18, 23, 67]. Variability in reported findings likely reflected differences in perioperative care pathways, outcome definitions, and patient populations.

Few studies employed standardized patient reported outcome measures, and reporting of analgesic use, pain scales, and recovery milestones remained inconsistent. Consequently, current evidence remains insufficient to clarify differences in patient centred recovery outcomes across minimally invasive approaches.

Recent studies evaluating robotic cholecystectomy have also reported concerns regarding bile duct injury during early phases of robotic adoption. Prolonged learning curve patterns associated with bile duct injury reporting during robotic cholecystectomy implementation were described in recent studies [88]. Higher adjusted bile duct injury rates in robotic cholecystectomy cohorts across multiple patient complexity strata were also reported in recent observational analyses [60]. These findings highlight the importance of training volume, institutional oversight, and implementation strategies during robotic adoption.

Beyond immediate perioperative morbidity, bile duct injury has also been associated with increased downstream healthcare utilization and payer level expenditures, reinforcing its relevance as an outcome measure in comparative evaluations of minimally invasive cholecystectomy [61].

### Oncological outcomes and surgical radicality

Available evidence described similar early oncological findings across robotic and laparoscopic approaches, including R0 resection rates and lymphadenectomy outcomes [28, 65, 66]. However, long term oncological outcomes, including recurrence and survival, were inconsistently reported across studies and remain limited in the current literature. Several studies discussed oncological outcomes in relation to anatomical planning, vascular control, and margin status rather than operative platform alone [17, 64]. In the context of biliary reconstruction, particularly for hilar cholangiocarcinoma, robotic platforms have been described in relation to dexterity and visualization during intracorporeal suturing and reconstruction. However, clinically relevant outcomes such as anastomotic leak rates, biliary stricture formation, and long-term biliary patency were inconsistently reported across studies.

Current evidence remains insufficient to clarify whether reported differences extend beyond technical feasibility. Reduced long term survival following bile leak or bile duct injury has also been described in population level analyses [89].

### Learning curve and cost considerations

Several studies described progressive changes in operative metrics during later phases of robotic adoption, particularly in studies involving surgeons with prior laparoscopic experience [40, 64, 90]. However, definitions of proficiency thresholds and learning curve endpoints varied substantially across studies.

Robotic surgery was frequently associated with higher procedural costs, largely related to platform acquisition, maintenance, and disposable instrumentation [17, 18]. Formal cost effectiveness analyses and incremental cost effectiveness ratios were not identified among included studies. Economic reporting varied substantially across studies, particularly regarding indirect institutional costs and downstream healthcare utilization.

### Limitations and future directions

The available evidence remains limited by the predominance of retrospective designs, selection bias, heterogeneity in outcome reporting, and concentration of robotic experience within high volume centres. Importantly, this review was not designed to establish causal superiority or comparative effectiveness between robotic and laparoscopic approaches. The findings should therefore be interpreted as descriptive and hypothesis-generating, given that most included studies were observational and subject to confounding by indication, surgeon expertise, institutional factors, and case selection.

Administrative databases often lack sufficient granularity to accurately capture intraoperative variables such as conversion to open surgery and may misclassify surgical approach. Important limitations in claims-based identification of robotic surgery have also been described in administrative database studies, further complicating interpretation of large database analyses [84].

The restriction to studies published in English and Spanish may have introduced language bias and limited the global representativeness of findings. Similarly, restriction to studies published from 2019 onwards was intended to reflect contemporary surgical practice and current generation robotic platforms, although this approach may have excluded earlier relevant studies.

Future research should prioritize prospective multicentre studies incorporating standardized complexity scoring systems, validated patient reported outcomes, long term oncological follow up, and formal cost effectiveness analyses to better characterize minimally invasive hepatobiliary surgery across different procedural settings.

## Conclusions

The available literature on robotic and laparoscopic hepatobiliary surgery remains heterogeneous in methodology, procedural classification, and outcome reporting. Across included studies, perioperative, recovery, and oncological findings varied according to procedural complexity, surgeon experience, institutional context, and study design.

Several studies reported lower intraoperative blood loss and fewer conversions to open surgery in robotic cohorts undergoing technically demanding procedures, whereas longer operative times were frequently reported in robotic surgery. However, the predominance of retrospective designs and heterogeneous outcome reporting limit interpretation of comparative findings.

Current evidence remains insufficient to establish comparative effectiveness or causal superiority between robotic and laparoscopic hepatobiliary surgery. Future prospective multicentre studies using standardized complexity frameworks and validated outcome measures are needed to better characterize minimally invasive hepatobiliary surgery across different procedural settings.

## Supplementary Information

Supplementary Material 1.

Supplementary Material 2.

## Data Availability

The data that support the findings of this study are derived from publicly available sources. All included studies are cited within the manuscript.
