# Supplementary material for: Robotic versus laparoscopic surgery in hepatobiliary procedures: a scoping review
Source: BMC Surg. 2026 Jul 4;26:640. doi: 10.1186/s12893-026-03907-y (PMC13613746; doi:10.1186/s12893-026-03907-y)
Supplement: Supplementary file 1 [file 12893_2026_3907_MOESM2_ESM.docx]

**SUPPLEMENTARY MATERIAL**

**Search Strategy**

| **Database** | **Search Algorithm** |
| --- | --- |
| **PubMed** | *(("Laparoscopy"[Mesh] OR "laparoscopic surgery"[Title/Abstract] OR laparoscopy[Title/Abstract])*  *AND ("Robotic Surgical Procedures"[Mesh] OR "robotic surgery"[Title/Abstract] OR "robot-assisted surgery"[Title/Abstract]))*  *AND ("General Surgery"[Mesh] OR "Colorectal Surgery"[Mesh] OR "Hernia, Abdominal"[Mesh] OR colectomy[Title/Abstract] OR gastrectomy[Title/Abstract] OR "colorectal surgery"[Title/Abstract] OR hepatectomy[Title/Abstract] OR "liver resection"[Title/Abstract] OR cholecystectomy[Title/Abstract] OR "pancreatic surgery"[Title/Abstract] OR pancreatectomy[Title/Abstract] OR "hernia repair"[Title/Abstract])*  *AND ("Treatment Outcome"[Mesh] OR "perioperative outcomes"[Title/Abstract] OR "postoperative recovery"[Title/Abstract] OR "postoperative complications"[Title/Abstract] OR "length of stay"[Title/Abstract] OR "operative time"[Title/Abstract] OR "blood loss"[Title/Abstract] OR "conversion to open surgery"[Title/Abstract] OR readmission[Title/Abstract] OR mortality[Title/Abstract] OR "quality of life"[Title/Abstract] OR "patient satisfaction"[Title/Abstract])* |
| **Scopus** | *TITLE-ABS-KEY("laparoscopic surgery" OR laparoscopy)*  *AND TITLE-ABS-KEY("robotic surgery" OR "robot-assisted surgery")*  *AND TITLE-ABS-KEY("abdominal surgery" OR colectomy OR gastrectomy OR "colorectal surgery" OR hepatectomy OR "liver resection" OR cholecystectomy OR "pancreatic surgery" OR pancreatectomy OR "hernia repair")*  *AND TITLE-ABS-KEY("perioperative outcomes" OR "postoperative recovery" OR "postoperative complications" OR "length of stay" OR "operative time" OR "blood loss" OR "conversion to open surgery" OR readmission OR mortality OR "quality of life" OR "patient satisfaction")* |
| **Web of Science** | *TS=("laparoscopic surgery" OR laparoscopy)*  *AND TS=("robotic surgery" OR "robot-assisted surgery")*  *AND TS=("abdominal surgery" OR colectomy OR gastrectomy OR "colorectal surgery" OR hepatectomy OR "liver resection" OR cholecystectomy OR "pancreatic surgery" OR pancreatectomy OR "hernia repair")*  *AND TS=("perioperative outcomes" OR "postoperative recovery" OR "postoperative complications" OR "length of stay" OR "operative time" OR "blood loss" OR "conversion to open surgery" OR readmission OR mortality OR "quality of life" OR "patient satisfaction")* |
